# Measurements of the Effective Stress Coefficient for Elastic Moduli of Sandstone in Quasi-Static Regime Using Semiconductor Strain Gauges

**DOI:** 10.3390/s24041122

**Published:** 2024-02-08

**Authors:** Vassily Mikhaltsevitch, Maxim Lebedev

**Affiliations:** 1 Centre for Exploration Geophysics, Curtin University, GPO Box U1987, Perth, WA 6845, Australia; v.mikhaltsevitch@curtin.edu.au; 2 Centre for Sustainable Energy and Resources, Edith Cowan University, 270 Joondalup Dr, Joondalup, WA 6027, Australia

**Keywords:** strain gauges, elastic properties, wave propagation, seismic frequency

## Abstract

Numerous experimental and theoretical studies undertaken to determine the effective stress coefficient for seismic velocities in rocks stem from the importance of this geomechanical parameter both for monitoring changes in rock saturation and pore pressure distribution in connection with reservoir production, and for overpressure prediction in reservoirs and formations from seismic data. The present work pursues a task to determine, in the framework of a low-frequency laboratory study, the dependence of the elastic moduli of n-decane-saturated sandstone on the relationship between pore and confining pressures. The study was conducted on a sandstone sample with high quartz and notable clay content in a quasi-static regime when a 100 mL tank filled with n-decane was directly connected to the pore space of the sample. The measurements were carried out at a seismic frequency of 2 Hz and strains, controlled by semiconductor strain gauges, not exceeding 10^−6^. The study was performed using a forced-oscillation laboratory apparatus utilizing the stress–strain relationship. The dynamic elastic moduli were measured in two sets of experiments: at constant pore pressures of 0, 1, and 5 MPa and differential pressure (defined as a difference between confining and pore pressures) that varied from 3 to 19 MPa; and at a constant confining pressure of 20 MPa and pore pressure that varied from 1 to 17 MP. It was shown that the elastic moduli obtained in the measurements were in good agreement with the Gassmann moduli calculated for the range of differential pressures used in our experiments, which corresponds to the effective stress coefficient equal to unity.

## 1. Introduction

The combined effect of confining stress and pore pressure on rock parameters is commonly described by the concept of an effective stress [1,2], which is fundamental to the study of fluid-saturated rock deformations under pressure. The knowledge of the effective stress for rock masses is of great significance to numerous geotechnical applications, including the quantitative interpretation of time-lapse seismic measurements [3,4], geothermal extraction [5], and seismic reservoir characterization [6].

In general, the effective stress concept considers rock properties as functions of a linear combination of confining stress and pore pressure presented by the tensor [7,8,9,10,11]:

(1) $$\sigma_{ij}^{eff}=\sigma_{ij}^{c}-n\delta_{ij}p,$$

Here, $\sigma_{ij}^{eff}$ are the effective stresses, $\sigma_{ij}^{c}$ are the total confining stresses, p is the pore pressure, and $\delta_{ij}$ is the Kronecker delta; n is the effective stress coefficient.

In recent decades, the concepts of the effective stress and effective stress coefficient have been subjects of intensive research of both theoretical (see, e.g., [12,13,14,15,16,17,18]) and experimental natures [7,14,19,20,21,22,23]. As was demonstrated in a number of studies [16,24,25,26], all rock properties cannot be described by a single effective stress coefficient, and different values of the coefficient should be introduced for different physical quantities. Furthermore, the values of the effective stress coefficients are dependent on the micro-heterogeneity of rocks, associated primarily with the rock mineral content and pore structure [14,16,18,26,27]. As was shown by Ciz et al. [27] and Glubokovskikh and Gurevich [18], the effective stress coefficient for bulk modulus of a rock is sensitive to the contrast between the moduli of the rock mineral constituents and can vary over a wide range. However, theoretical analyses by Gardner et al. [14], Gurevich [17], and Pride [28] demonstrate that in the case of a linearly elastic micro-homogeneous grain material, the effective stress coefficient for the elastic moduli is equal to unity. Since the same micro-homogeneous material was used in the Gassmann theory [29], the validity of such analyses, as pointed out by Berryman [16], is based on an implicit assumption that just as a natural rock can be satisfactorily substituted by an equivalent homogeneous rock in the Gassmann model, it can also be substituted in a similar way in the effective stress analytical model. When such a substitution is possible, we can expect that the effective stress coefficients are equal to unity for all the scale-invariant physical quantities (i.e., quantities dependent on the pore space geometry and grain material moduli, but not dependent on the absolute value of the pore space), including porosity, as well as the low- and high-frequency limits of the poroelastic moduli [16,28].

However, despite the similarity in approach to the Gassmann theory, which is well confirmed by numerous experiments (see, e.g., the recent review by Sevostianov [30]), the effective stress model based on the equivalent homogeneous rock substitution has not found significant support in most published experimental studies, and, in particular, in studies devoted to measuring the effective stress coefficient for elastic moduli or velocities [20,21,22,31].

On the other hand, it has to be noted that the great majority of laboratory measurements presented in literature are performed at ultrasonic frequencies under conditions of an uncontrolled level of dynamic strain in tested specimens [32]. As was demonstrated in a number of studies [33,34,35], the inelasticity of such a complex material as rock is a strain-dependent parameter controlled by internal friction inside the crystalline body of the rock. The main sources of the internal friction resulting from sliding interfaces that are in contact within the rock [33,34], and dislocation-breakaway damping [33], become negligible if amplitudes of strains caused by acoustic waves are less than 10^−6^. Such a strain amplitude limit better corresponds to the real conditions of seismological measurements and imposes important restrictions on experimental measurements of the elastic/anelastic properties of rocks in the laboratory. However, due to the complexity of the technical implementation of the strain-level control in ultrasonic systems, most ultrasonic measurements are performed without strain-amplitude control, which often leads to inadequate values of the measured elastic moduli [36].

To our knowledge, the only effective stress study at the strain level below 10^−6^ was conducted at a low frequency on Savonnieres limestone for the effective pressure coefficient for porosity by Tan et al. [23]. Analyzing the drained-to-undrained transition of bulk modulus in n-decane-saturated Savonnieres limestone observed in forced-oscillation experiments with varying dead volume, Tan et al. [23]. demonstrated that the prediction of the poroelastic model is highly consistent with the experimental data if the effective stress coefficient for porosity is equal to unity.

The goal of the present paper is to determine the stress-effective coefficient for elastic moduli of a polymineral sandstone sample at a seismic frequency with dynamic strains controlled throughout the entire experiment by semiconductor strain gauges at a level not exceeding 10^−6^. In addition to the predominant mineral presented by quartz, the sample selected for our measurements (Mungaroo Formation, Western Australia) contains a number of other minerals, including a significant amount of clay minerals. We present the results of the laboratory measurements of the elastic properties of rock performed using a forced-oscillation apparatus at a frequency of 2 Hz. Using the standard low-frequency forced-oscillation method (a comprehensive review of the forced-oscillation method is presented in [32]), we examined the dependences of the elastic moduli of the sandstone and extensional attenuation on the differential pressure, which is defined as the difference between confining and pore pressures, and determined the effective stress coefficient for elastic moduli. To avoid lacerations of strain gauges due to the high compressibility of the sample, we had to limit our measurements to an upper confining pressure of 20 MPa. The measurements were carried out in two sets. The first set of the measurements was conducted at pore pressures equal to 0, 1, and 5 MPa and a differential pressure gradually increasing from 3 MPa to 19 MPa, and the second set was performed at a constant confining pressure of 20 MPa and a pore pressure varying from 1 to 17 MPa; in both sets of the measurements, n-decane was used as the pore fluid. In all measurements, the dynamic strains did not exceed 10^−6^.

## 2. Experimental Setup

In this study, experiments at seismic frequencies were performed using a low-frequency laboratory apparatus with the longitudinal type of the forced oscillations described in detail by Mikhaltsevitch et al. [32]. The apparatus operates at frequencies of 0.01 Hz to 100 Hz and strain amplitudes of 10^−8^ to 10^−6^. The apparatus measures the elastic parameters of rock samples at confining or uniaxial pressures from 0 to 70 MPa.

**Figure 1:**
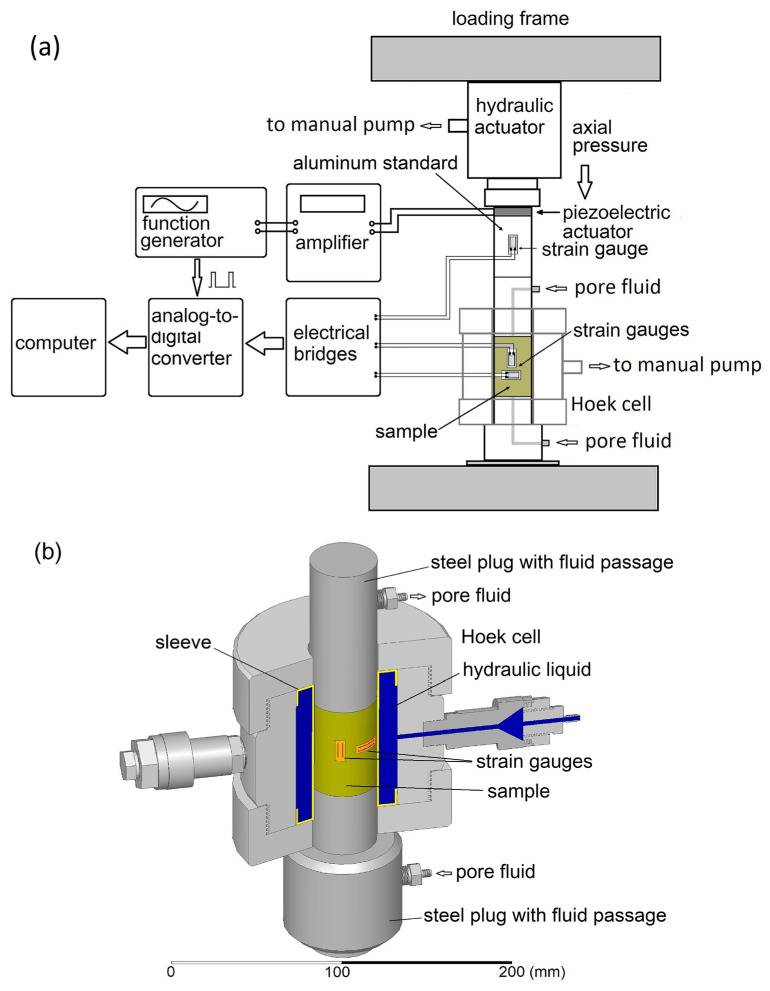
The mechanical assembly and electrical schematics of the low-frequency laboratory apparatus (**a**); the measured sample in the Hoek cell with two strain gauges mounted along the axis and circumference of the sample (**b**).

The mechanical assembly and electrical schematic of the low-frequency setup are presented in Figure 1a. The assembly consists of a steel frame and a column of units located in the center of the frame. The column includes a hydraulic actuator, a Hoek triaxial cell (model 45-D0554, Controls Group), a piezoelectric stack actuator P-035.10P (Physik Instrumente GmbH & Co. KG, Karlsruhe, Germany), an aluminum standard, and two steel cylinders with fluid passages. A measured sample is inserted inside an elastomer sleeve located at the center of the Hoek cell. The confining pressure is provided by two manual hydraulic pumps (model P392, Enerpac, Milwaukee, WI, USA) linked to the cell and hydraulic actuator (model RCS201, Enerpac), which apply lateral and axial stresses to the sample. The dynamic strains caused by the piezoelectric actuator are registered by three strain gauges (KSP-6-350-E4, Kyowa Ltd., Novi, MI, USA). One gauge is attached to the standard and the other two are attached to the sample.

The strain gauge on the aluminum standard is orientated in the axial direction, and the two strain gauges attached to the test sample are orientated along the axis and circumference of the sample (Figure 1b). Through a set of electric bridges (BCM-1 Wheatstone Bridge, Omega Engineering Ltd., Manchester, UK), the strain gauges are connected with an analog-to-digital converter (model 100, InstruNet, Omega Engineering, Norwalk, CT, USA). The electrical connection of the gauges on the specimen is implemented with a feedthrough assembly (Spectite WFS, Hillside, IL, USA).

The multilayer piezoelectric actuator transforms the periodic voltage, applied by a function generator via a power amplifier, into mechanical stress causing dynamic modulation of the electric resistance of the strain gauges. Electrical bridges convert modulated resistance into voltage oscillations, which, after digitization via an analog-to-digital converter, is received by a computer for further processing.

Since voltage oscillations contain information about the amplitudes of the corresponding strains, we can find Poisson’s ratio ν and Young’s modulus E as below:

(2) $$E=E^{st}\frac{\varepsilon_{ax}^{st}}{\varepsilon_{ax}}$$

(3) $$\nu =\frac{\varepsilon_{rad}}{\varepsilon_{ax}}$$

After finding E and ν, the bulk K and shear μ moduli can be found as follows:

(4) $$K=\frac{E}{3(1-2\nu )}$$

(5) $$\mu =\frac{E}{2\left(1+\nu \right)}$$

The extensional attenuation $Q_{E}^{-1}$ in the sample is derived from the phase shift Δφ between the harmonic stress applied to the sample and resulting strain detected in the sample [32]:

(6) $$Q_{E}^{-1}=\Delta \varphi$$

The low-frequency measurements presented below were conducted at an oscillating frequency of 2 Hz. During the measurements, the stress–strain readings for every 100 oscillations were averaged to improve the signal-to-noise ratio of the signals obtained from strain gauges. The uncertainty of the extensional attenuation measurements was ±0.003.

## 3. Sample Description and Experimental Procedure

The sandstone sample measured in the present study was retrieved from the Triassic Mungaroo Formation, Western Australia. Its mineral composition, obtained using X-ray fluorescence and diffraction analysis, is presented in Table 1. Cementation of the framework grains in the Mungaroo sandstone is mainly associated with the presence of quartz overgrowths and kaolinite [37].

**Figure 2:**
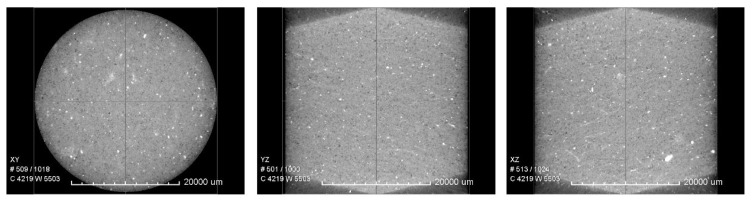
Two-dimensional mutually perpendicular X-ray cross-sections of the Mungaroo sandstone sample.

The homogeneity of the sample was verified using an X-ray microscope, VersaXRM-500, Xradia Ltd., Pleasanton, CA, USA (Figure 2). Based on the obtained X-ray images, we assume that the sandstone sample tested in this study is homogeneous on a macroscopic (gauge-length size) scale.

The physical parameters of the sample are as follows. The length of the test sample is equal to 75.2 mm and the diameter is 37.8 mm. The mass and density of the dry/wet sample are 190.48/198.9 g and 2268/2368 kg/m^3^, respectively. The difference in density of the sample in dry and wet states corresponds to a pore space of 11.5 cm^3^ or porosity of 13.7%. The permeability of the sandstone is equal to 1.9 mD.

The experimental procedure was organized in the following way. For avoiding the influence of moisture trapped between contacts, which can be significant in sandstones [38], prior to the measurements, the sample was vacuum-dried in an oven at a temperature of ~60 °C. Then, the sample was placed in the elastomer sleeve mounted inside the triaxial cell, where it was saturated with n-decane at a confining pressure of 10 MPa. To ensure the full saturation, at least five pore volumes of liquid were pumped through the sample. The flow rate of liquid during saturation did not exceed 0.02 cm^3^/s (1.2 cm^3^/min).

**Table 1:** The petrographic data for the Mungaroo sandstone sample.

Mineral	Content (Volume), %	Bulk Modulus, GPa
Quartz	72	36.6 [39]
K-feldspar	2	57 [40]
Micrite	4	71 [41]
Illite	5	21 [42]
Kaolinite	12	11 [43]
Calcite	4	76.8 [39]

**Figure 3:**
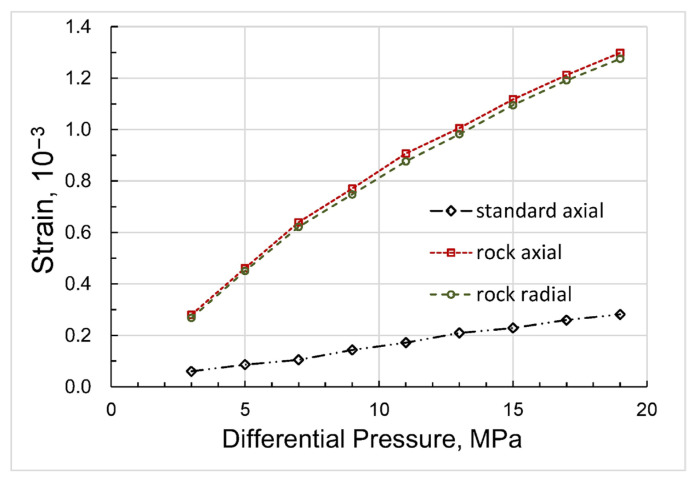
The dependences of static strains in the sample and standard on confining pressure measured in the drained regime.

After saturation, we studied the pressure dependences of the elastic and anelastic parameters, such as Young’s modulus, bulk and shear moduli, Poisson ratio, and extensional attenuation, on confining pressure at seismic frequency of 2 Hz in the drained regime when the fluid line connected to the top end of the sample was open. The measurements of the static strains in the sample and standard during this experiment (see Figure 3) demonstrate that the changes in radial and axial strains in response to an increase in confining pressure are virtually equivalent, which also confirms the homogeneity of the sample. The uncertainty of the strain measurements is mainly determined by the uncertainty of the strain-gauge factor and was equal to ±3%. Then, the same measurements as in the drained regime were repeated at pore pressures equal successively to 1 and 5 MPa when the 100 mL tank of the syringe pump (model 100DX, Teledyne ISCO, Lincoln, NE, USA) filled with n-decane was directly connected to the pore space of the sample to minimize pore pressure fluctuations and their effect on the grain contacts in the sample during the experiment. Finally, we conducted the last set of measurements when the confining pressure was fixed at the level of 20 MPa and pore pressure controlled using the syringe pump was gradually increasing from 1 MPa to 17 MPa. The accuracy of the obtained experimental data was directly dependent on the uncertainty of the dynamic strain measurements, which was mainly determined by the uncertainty of the strain-gauge factor and equal to ±3%.

Let us also demonstrate here that all measurements carried out in our study can be classified as quasi-static measurements. According to the definition of the quasi-static process (see, e.g., [39]), the quasi-static process is a slow dynamic process compared to the relevant relaxation times (i.e., the times required for the system to reach equilibrium) that virtually presents a sequence of equilibrium states. The characteristic relaxation time for heterogeneous pore pressures of the sample size L is [39]:

(7) $$\tau =\frac{D}{L^{2}}$$

Here, $D=\frac{kK_{f}\phi }{\eta }$ is the fluid pressure diffusivity, $K_{f}$ = 1.15 GPa is bulk modulus of n-decane [44], η= 0.92 mPa∙s is the viscosity of n-decane [44], k = 1.9 mD is the permeability of the sample, and ϕ= 0.137 is the sample porosity. Therefore, at a frequency of the forced oscillations f≪1/τ, the pore pressure gradients caused by these oscillations have time to relax and reach equilibrium. For the tested sample, the characteristic frequency corresponding to the relaxation time τ is $f_{C}=\frac{1}{\tau }=$ 57 Hz. Thus, since the frequency of the forced oscillations in our experiments was equal to 2 Hz, we can consider our measurements as measurements conducted in the quasi-static regime.

## 4. Results and Discussion

### 4.1. Measurements with Variable Confining Pressure

In the experiments with variable confining pressure, the effective stress coefficient for the elastic moduli of the Mungaroo sandstone sample was estimated through a comparison of the elastic moduli obtained in the experiments with constant non-zero pore pressures with the elastic moduli obtained in the drained regime, which were considered as reference parameters in our study. The experiments with constant non-zero pore pressures included the measurements with two pore pressures of 1 and 5 MPa conducted under the differential pressure corresponding to the confining pressure of the drained regime, which was varying from 3 to 19 MPa.

**Figure 4:**
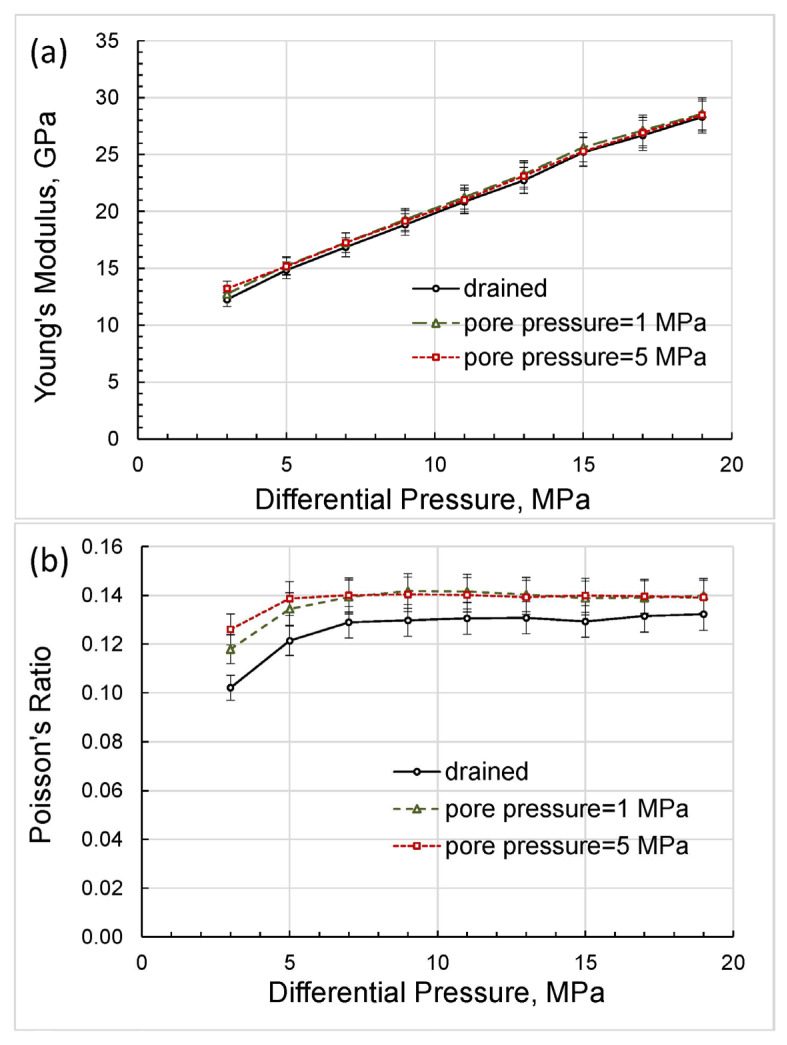
The dependences of Young’s modulus (**a**) and Poisson’s ratio (**b**) on differential pressure, measured on the Mungaroo sandstone sample at a frequency of 2 Hz.

**Figure 5:**
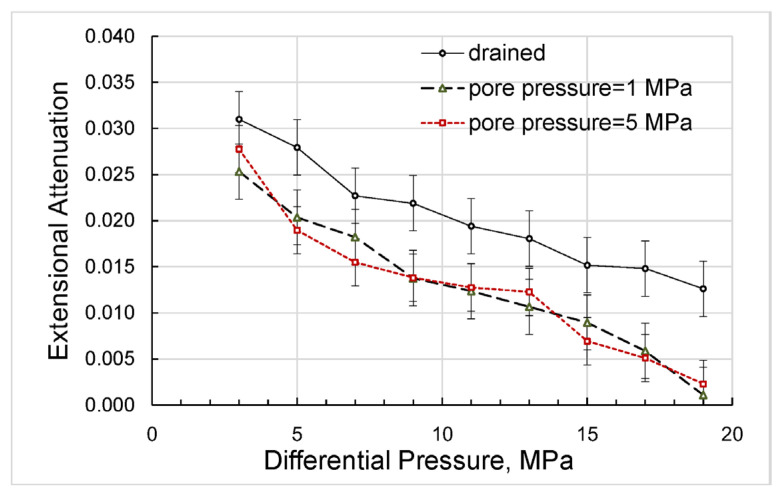
The dependence of the extensional attenuation on differential pressure, measured on the Mungaroo sandstone sample at a frequency of 2 Hz.

**Figure 6:**
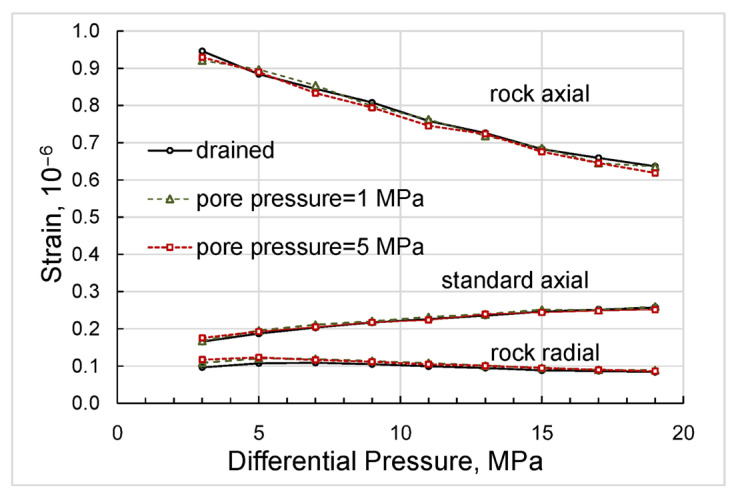
The dependences of the dynamic strains measured on the sample and standard on differential pressure, obtained for the Mungaroo sandstone sample at a frequency of 2 Hz.

The direct results of our measurements can be presented as differential-pressure dependences obtained for Young’s modulus and Poisson’s ratio (Figure 4), extensional attenuation (Figure 5), and dynamic strains (Figure 6) in the standard and rock. The measurement uncertainties given in the graphs were estimated according to the uncertainty analysis procedure developed for the forced-oscillation measurements by Adam et al. [45] and Adam et al. [46].

**Figure 7:**
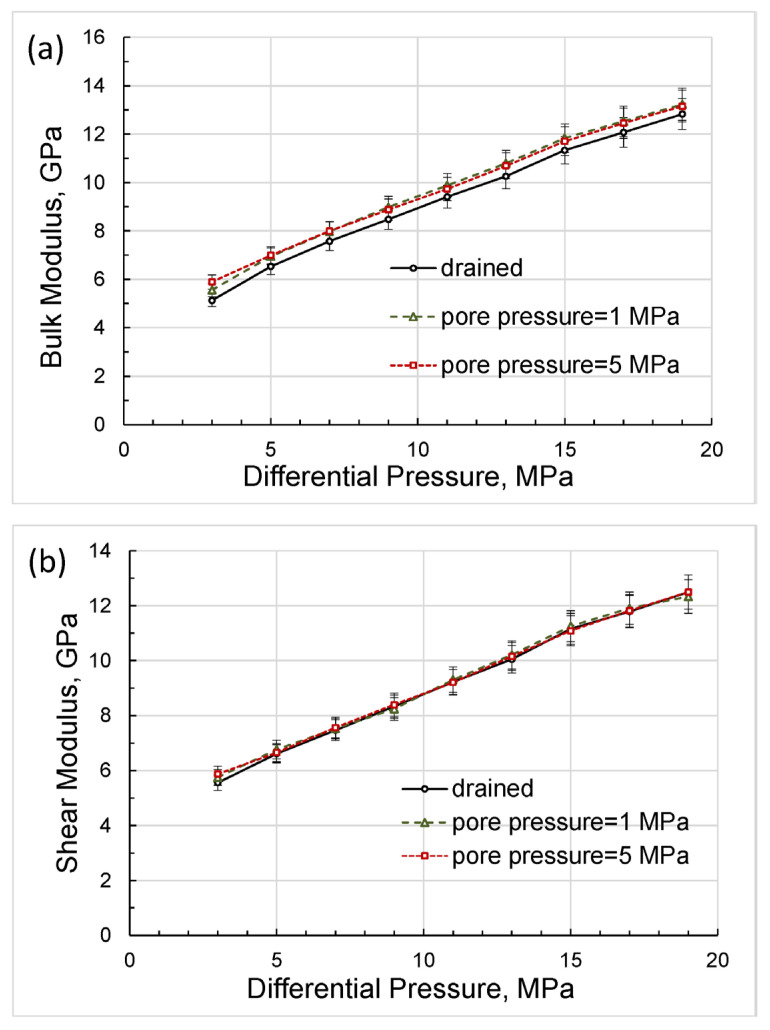
The dependences of the bulk modulus (**a**) and shear modulus (**b**) on differential pressure, computed using Equations (4) and (5).

The bulk and shear moduli calculated in accordance with Equations (4) and (5) are shown in Figure 7. The slight discrepancy between the drained bulk modulus and bulk moduli measured at pore pressures of 1 and 5 MPa is due to the finite size of the syringe-pump tank, which can be verified using the modified Gassmann model considering a dead volume, i.e., a pore-fluid volume external to the test sample [32].

The mineral bulk modulus of the sample can be computed as the Voigt-Reuss-Hill average [39]:

(8) $$K_{s}=\frac{K_{v}+K_{r}}{2}$$

where,

(9) $$K_{v}=\sum_{i=1}^{N}f_{i}K_{i}\;\mathrm{and}\;K_{r}=1/\sum_{i=1}^{N}\frac{f_{i}}{K_{i}}$$

Here, $f_{i}$ is the volume fraction of the *i*-th component of the solid phase of the rock and $K_{i}$ is the bulk modulus corresponding to this component.

Using the mineral bulk moduli of the Mungaroo sandstone presented in Table 1, we find:

(10) $$K_{s}=33.1\;\mathrm{GPa}$$

The bulk modulus of the n-decane-saturated sample can be found using the following modified Gassmann equation [47]:

(11) $$K_{sat}=K_{d}+\frac{{\left(1-\frac{K_{d}}{K_{s}}\right)}^{2}}{\frac{\alpha }{K_{f}}+\frac{1-\phi }{K_{s}}-\frac{K_{d}}{K_{s}^{2}}}$$

Here, $K_{d}$ is the drained bulk modulus, $\alpha =\phi +V_{D}/V_{s}$, and $V_{D}$ and $V_{s}$ are the dead volume and sample volume, respectively.

**Figure 8:**
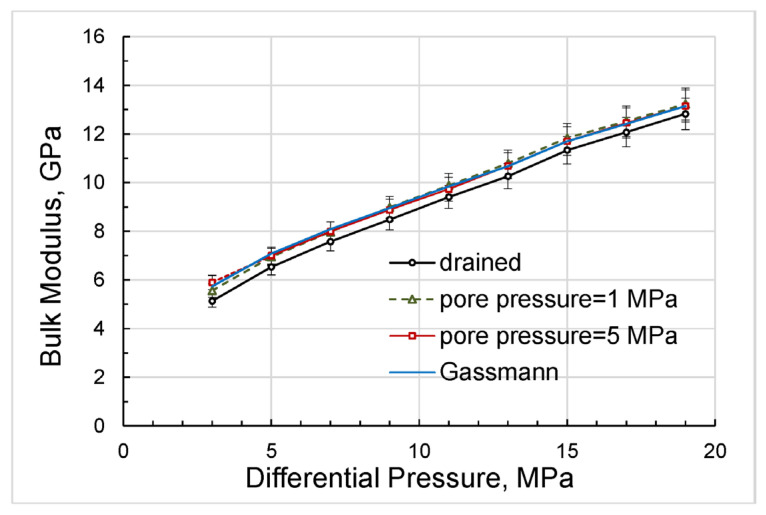
The experimental dependences of the bulk moduli on differential pressure, obtained using Equation (4), and the pressure dependence of the bulk modulus, computed using modified Gassmann Equation (11).

A comparison of the Gassmann moduli computed for the drained moduli obtained at differential pressures of 3 to 17 MPa with bulk moduli measured at non-zero constant pore pressures is presented in Figure 8.

As can be seen from Figure 8, there is good agreement between the experimental moduli with non-zero pore pressures and the Gassmann modulus.

### 4.2. Measurements with Variable Pore Pressure

**Figure 9:**
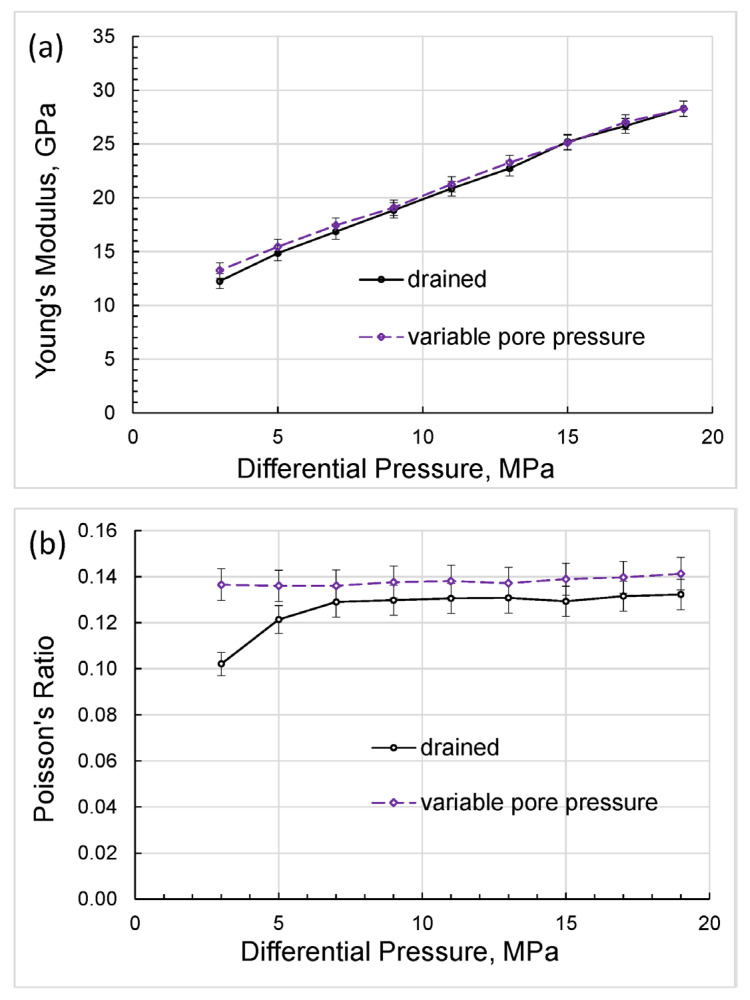
The dependences of the Young’s modulus (**a**) and Poisson’s ratio (**b**) on differential pressure, measured on the n-decane-saturated Mungaroo sandstone sample at a frequency of 2 Hz under a confining pressure of 20 MPa and a pore pressure varied that from 1 to 17 MPa.

**Figure 10:**
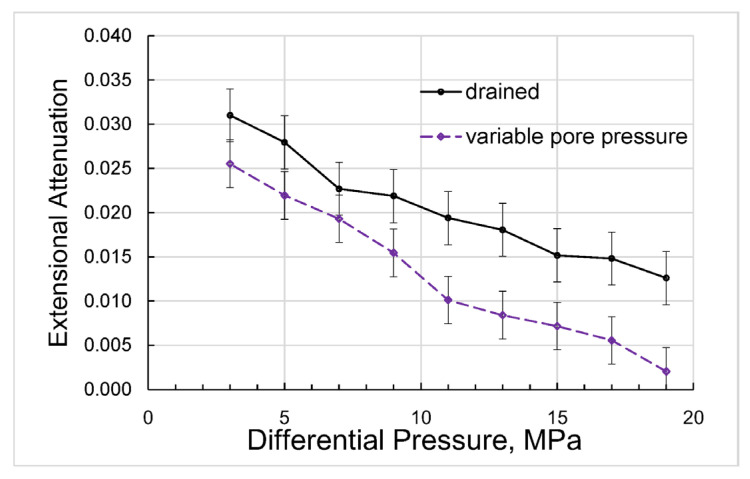
The dependence of the extensional attenuation on differential pressure, measured on the n-decane-saturated Mungaroo sandstone sample at a frequency of 2 Hz and presented with the attenuation obtained in the drained mode.

**Figure 11:**
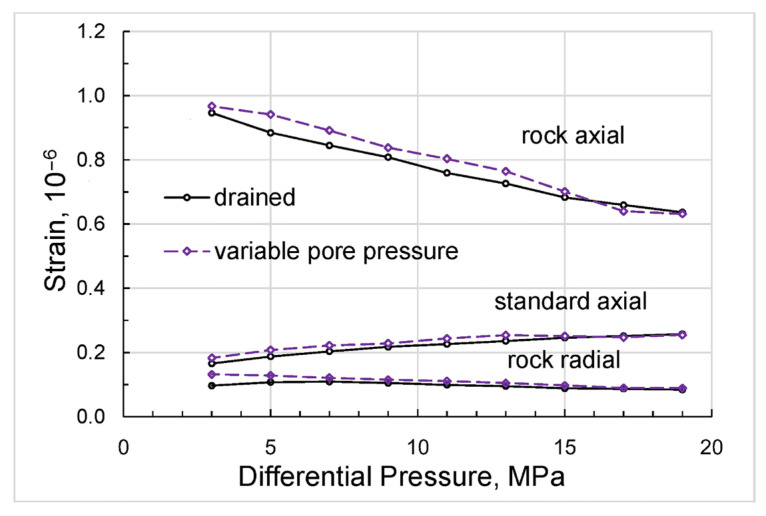
The differential pressure dependences of the dynamic strains measured on the n-decane-saturated Mungaroo sandstone sample and standard at a frequency of 2 Hz.

**Figure 12:**
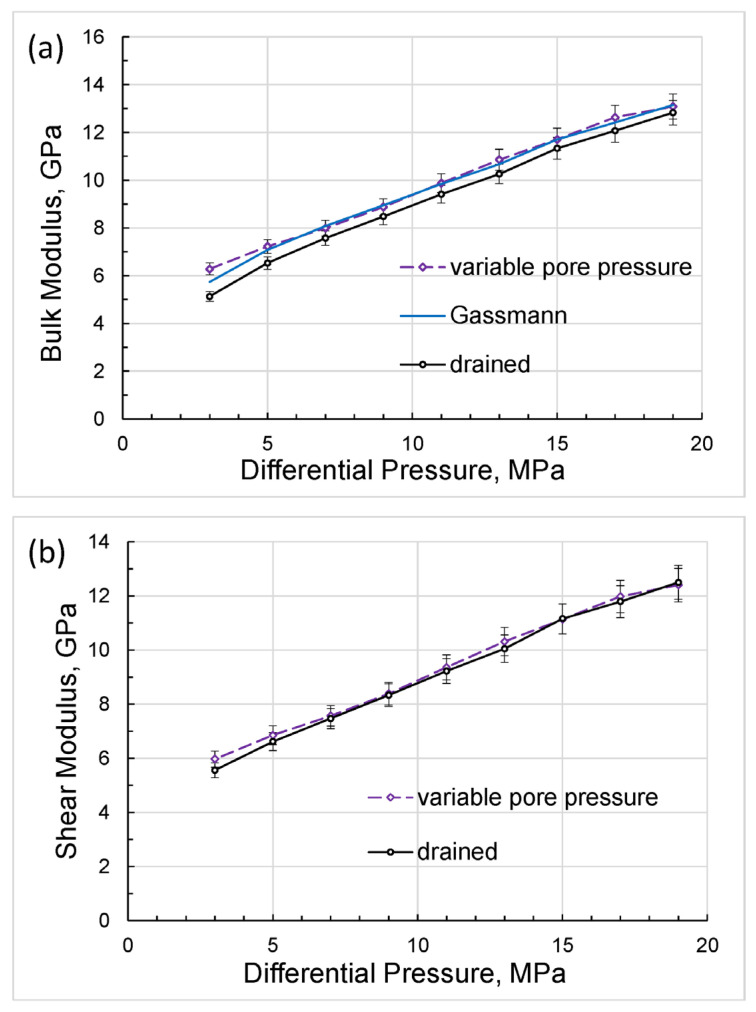
The dependences of the bulk modulus (**a**) and shear modulus (**b**) on differential pressure, calculated from measured Young’s modulus and Posson’s ratio.

The second series of experiments conducted in this study included the measurements performed at 2 Hz under a confining pressure of 20 MPa and a pore pressure raising gradually from 1 to 17 MPa. Note that the order of measurements corresponds to the reverse order of increasing values along the differential pressure axis. The direct results of these measurements are shown in Figure 9, Figure 10 and Figure 11 together with the data obtained in the drained experiment. The bulk and shear moduli calculated in accordance with Equations (3) and (4) are given in Figure 12.

### 4.3. Discussion

**Figure 13:**
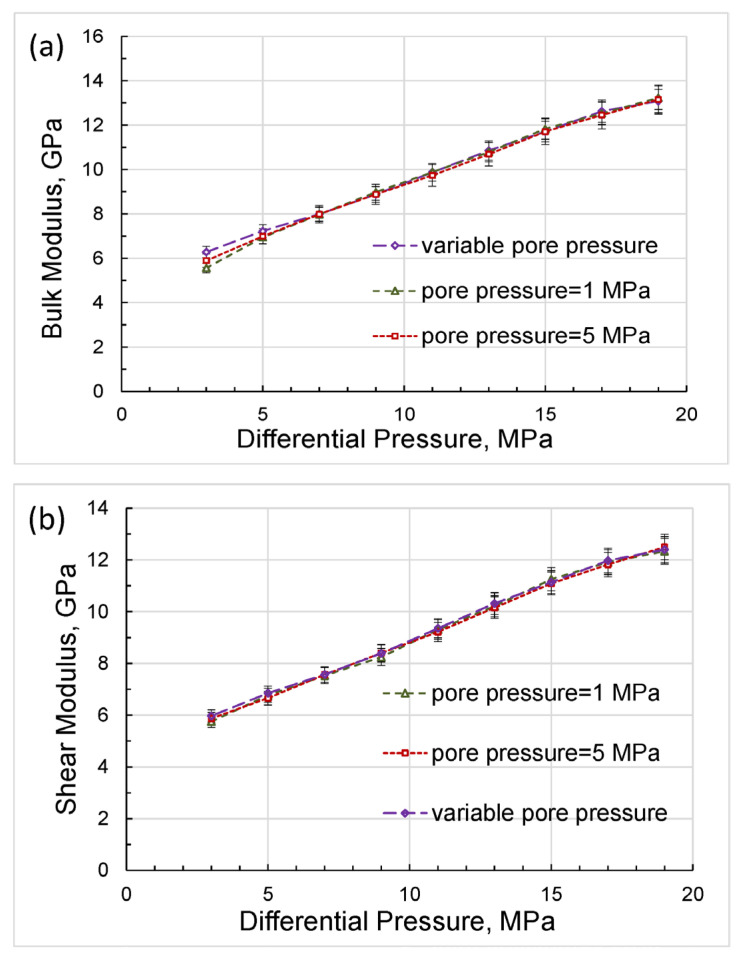
The dependences on differential pressure obtained for the bulk modulus (**a**) and shear modulus (**b**) in the measurements with variable and constant pore pressures.

**Figure 14:**
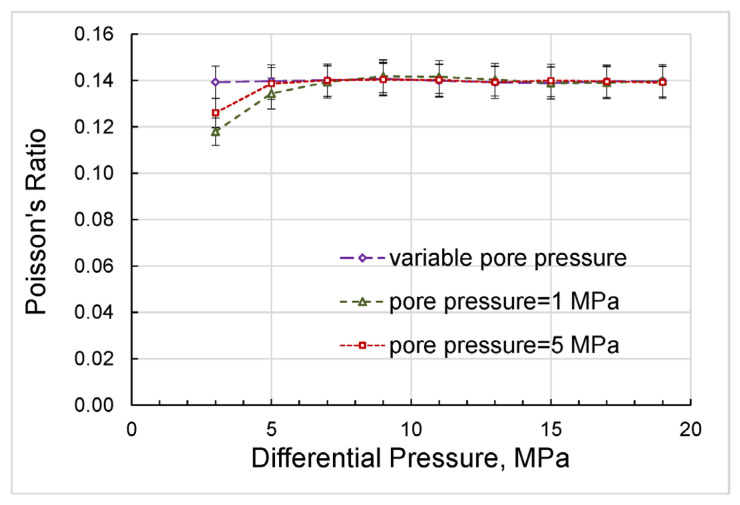
The dependences on differential pressure obtained for Poisson’s ratio in the measurements with variable and constant pore pressures.

The bulk/shear moduli and Poisson’s ratio obtained in the first set of the experiments at pore pressures of 1 and 5 MPa and in the second set of the experiments with variable pore pressure are compared in Figure 13 and Figure 14.

As follows from the pressure dependences presented in Figure 13, the bulk and shear moduli do not depend on the measurement technique and are uniquely determined as functions of the differential pressure, which coincides with the effective stress in the case when the effective stress coefficient is equal to unity.

The good agreement of the experimental elastic moduli with the Gassmann moduli, obtained in the range of differential pressures used in this study, with the exception of minor deviations at lower pressure values, indicates that the material of the studied rock can be satisfactorily substituted in the Gassmann model by an equivalent homogeneous material for each differential pressure. The dependences of the elastic moduli on pressure shown in Figure 13 convincingly demonstrate that the real rock material cannot be considered linearly elastic. However, at each point of differential pressure, the real rock can be replaced by an equivalent homogeneous rock of the same porosity that satisfies the Gassmann model but whose elastic properties differ from the properties of equivalent rocks at other pressures.

Let us now analyze the discrepancy between the bulk moduli that occurs at lower differential pressures of 3 to 7 MPa (Figure 13a) and reaches a maximum of 6 percent at a differential pressure of 3 MPa for bulk moduli measured with variable pore pressure and with a pore pressure equal to 1 MPa. This discrepancy can be explained as follows. First, note that the experiments with variable pore pressure started from the higher differential pressures and finished at lower differential pressures whereas the experiments with the constant pore pressures were carried out in the reversed order. Due to the duration of the low-frequency measurements, which lasted, as a rule, for many hours, the sample was experiencing creep under stress for a much longer period of time before the measurements at lower pressures began than in the first set of experiments. The long-time creep minimized dislocations, closed microcracks, and made stress in the pores more uniform, which increased the measured moduli. Generally, the difference in bulk moduli obtained in the two sets of the experiments was similar to the difference in drained bulk moduli measured under loading and unloading conditions by Pimienta et al. [48], where the variation between loading/unloading moduli reached more than 30% in Fontainebleau sandstone at differential pressures of ≤8 MPa.

As can be seen from Figure 14, the Poisson’s ratios obtained at constant pore pressures of 1 and 5 MPa display a sharp rise with differential pressure whilst the Poisson’s ratio measured at the variable pore pressure remains virtually pressure-independent. The latter confirms that the state of the solid framework of the rock at the lower pressures was not as different from that at higher pressures during the experiments with variable pore pressure as in the experiments with constant pore pressures. Note also that the bulk moduli and Poisson’s ratios obtained in both series of experiments converged significantly with increasing pressure, as shown in Figure 13a and Figure 14.

According to Equations (3) and (4), the effect of changing the Poisson’s ratio has a much smaller effect on the change in shear modulus than on the change in bulk modulus, which is confirmed by the negligible discrepancy of shear moduli at lower differential pressures, as can be observed in Figure 13b.

**Figure 15:**
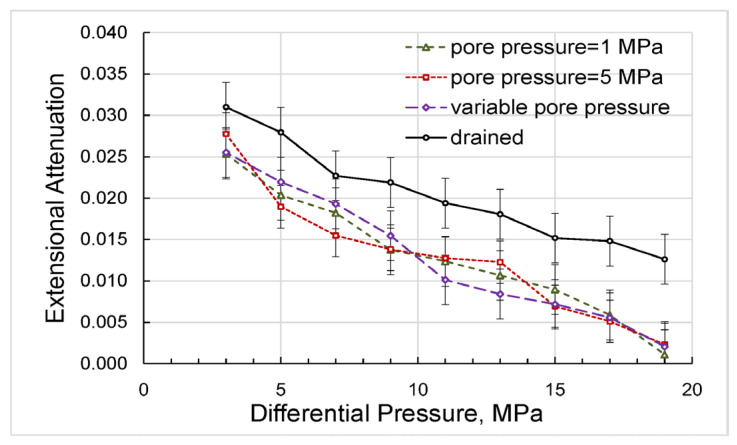
The dependences on differential pressure obtained for the extensional attenuation in the experiments with variable and constant pore pressures.

Let us point out that the dependences of the extensional attenuation on differential pressure obtained in both sets of experiments with non-zero pore pressures and presented in Figure 15 are also in a good match, which demonstrates that these dependences are functions of the effective stress with the effective stress coefficient equal to unity (see Equation (1)). The difference between the dependences corresponding to the drained and undrained regimes can be attributed to friction caused by n-decane moving freely in and out of the sample to ensure constant zero pore pressure under the applied forced oscillations.

## 5. Conclusions

We have presented the results of the laboratory study carried out to investigate the dependence of the elastic moduli of fluid-saturated sandstone on the relationship between pore and confining pressures. The study was conducted on a sandstone sample with high quartz and substantial clay content saturated with n-decane, where a tank whose volume was approximately equal to 10 times the size of the pore volume, filled with n-decane, was directly connected to the pore space of the sample to smooth out pore pressure variations. The experiments were carried out in the quasi-static regime at a seismic frequency of 2 Hz using a forced-oscillation laboratory apparatus based on a longitudinal type of forced oscillations. The strains in the sample were controlled using semiconductor strain gauges and maintained at a level not exceeding 10^−6^. The dynamic elastic moduli were measured in the drained regime with zero pore pressure, as well as in the experiments with non-zero constant pore pressures of 1 and 5 MPa at a differential pressure that varied from 3 to 19 MPa, and with pore pressure changed from 1 to 17 MP at a constant confining pressure of 20 MPa. It was shown that the measurement results of elastic moduli were in good agreement with the Gassmann moduli calculated for the range of differential pressure used in this study, which corresponds to the effective stress coefficient equal to unity. It was also demonstrated that the results of the measurements of extensional attenuation obtained at non-zero pore pressures are in good agreement with each other and are uniquely determined as functions of differential pressure.

## Data Availability

The data that support the findings of this study are openly available in the Zenodo repository at https://doi.org/10.5281/zenodo.10408847.

## References

[1] Terzaghi K. (1943). Theoretical Soil Mechanics.

[2] Skempton A.W. (1960). Effective stress in soils, concrete and rock. Select. Pap. Soil. Mech..

[3] Landrø M. (2001). Discrimination between pressure and fluid saturation changes from time lapse seismic data. Geophysics.

[4] Vasco D.W. (2004). Seismic imaging of reservoir flow properties: Time-lapse pressure changes. Geophysics.

[5] Chen S., Zhao Z., Chen Y., Yang Q. (2020). On the effective stress coefficient of saturated fractured rocks. Comput. Geotech..

[6] Avseth P., Mukerji T., Mavko G. (2005). Quantitative Seismic Interpretation: Applying Rock Physics Tools to Reduce Interpretation Risk.

[7] Nur A., Byerlee J.D. (1971). An exact effective stress law for elastic deformation of rock with fluids. J. Geophys. Res..

[8] Carroll M.M. (1979). An effective stress law for anisotropic elastic deformation. J. Geophys. Res..

[9] Thompson M., Willis J.R. (1991). A reformation of the equations of anisotropic poroelasticity. J. Appl. Mech..

[10] Detournay E., Cheng A.H.D., Fairhurst C. (1993). Fundamentals of poroelasticity. Comprehensive Rock Engineering: Principles, Practice and Projects, Volume 2, Analysis and Design Method.

[11] Cheng A.H.-D. (1997). Material coefficients of anisotropic poroelasticity. Int. J. Rock Mech. Min. Sci..

[12] Biot M.A., Willis D.G. (1957). The elastic coefficients of the theory of consolidation. J. Appl. Mech..

[13] Geertsma J. (1957). The effect of fluid pressure decline on volumetric changes of porous rocks. Petrol. Trans. AIME.

[14] Gardner G.H.F., Wyllie M.R.J., Droschak D.M. (1965). Hysteresis in the velocity-pressure characteristics of rocks. Geophysics.

[15] Zimmerman R.W., Somerton W.H., King M.S. (1986). Compressibility of porous rocks. J. Geophys. Res..

[16] Berryman J.G. (1992). Effective stress for transport properties of inhomogeneous porous rock. J. Geophys. Res..

[17] Gurevich B. (2004). A simple derivation of the effective-stress coefficient for seismic velocities in porous rocks. Geophysics.

[18] Glubokovskikh S., Gurevich B. (2014). Effect of micro-inhomogeneity on the effective-stress coefficients and undrained bulk modulus of a poroelastic medium: A double spherical shell model. Geophys. Prospect..

[19] Todd T., Simmons G. (1972). Effect of pore pressure on the velocity of compression waves in low-porosity rocks. J. Geophys. Res..

[20] Christensen N.I., Wang H.F. (1985). The Influence of pore pressure and confining pressure on dynamic elastic properties of Berea sandstone. Geophysics.

[21] Prasad M., Manghnani M. (1997). Effects of pore and differential pressure on compression wave velocity and quality factor in Berea and Michigan sandstones. Geophysics.

[22] Vasquez G.F., Vargas E.A., Ribeiro C.J.B., Leao M., Justen J.C.R. (2009). Experimental determination of the effective pressure coefficients for Brazilian limestones and sandstones. Rev. Bras. Geof..

[23] Tan W., Müller T.M., Ba J., Mikhaltsevitch V., Cao C. (2020). Drained-to-undrained transition of bulk modulus in fluid-saturated porous rock induced by dead volume variation. Geophys. Prospect..

[24] Robin P.-Y.F. (1973). Note on effective pressure. J. Geophys. Res..

[25] Carroll M.M., Katsube N. (1983). The role of Terzaghi effective stress in linearly elastic deformation. J. Energy Resour. Technol..

[26] Zimmerman R.W. (1991). Compressibility of Sandstones.

[27] Ciz R., Siggins A.F., Gurevich B., Dvorkin J. (2008). Influence of microheterogeneity on effective stress law for elastic properties of rocks. Geophysics.

[28] Pride S.R., Rubin Y., Hubbard S. (2005). Relationships between seismic and hydrological properties. Hydrogeophysics.

[29] Gassmann F. (1951). Über die elastizität poröser medien. Vierteljahrsschr. Naturforsch. Ges. Zür..

[30] Sevostianov I. (2020). Gassmann equation and replacement relations in micromechanics: A review. Int. J. Eng. Sci..

[31] Njiekak G., Schmitt D.R. (2019). Effective-stress coefficient for seismic velocities in carbonate rocks: Effects of pore characteristics and fluid types. Pure Appl. Geophys..

[32] Mikhaltsevitch V., Lebedev M., Chavez R., Vargas E.A., Vasquez G.F. (2021). A laboratory forced-oscillation apparatus for measurements of elastic and anelastic properties of rocks at seismic frequencies. Front. Earth Sci..

[33] Gordon R.B., Davis L.A. (1968). Velocity and attenuation of seismic waves in imperfectly elastic rock. J. Geophys. Res..

[34] Mavko G. (1979). Frictional attenuation: An inherent amplitude dependence. J. Geophys. Res. Solid Earth.

[35] Winkler K., Nur A., Gladwin M. (1979). Friction and seismic attenuation in rocks. Nature.

[36] Nourifard N., Lebedev M. (2019). Research note: The effect of strain amplitude produced by ultrasonic waves on its velocity. Geophys. Prospect..

[37] Jong J.T. (1996). Sedimentary History, Diagenesis and Organic Facies of the Triassic Mungaroo Formation, Barrow Sub-Basin, WA. Master’s Thesis.

[38] Murphy W.F., Winkler K.W., Kleinberg R.L. (1984). Frame modulus reduction in sedimentary rocks: The effect of adsorption on grain contacts. Geophys. Res. Lett..

[39] Mavko G., Mukerji T., Dvorkin J. (2020). The Rock Physics Handbook.

[40] Angel R.J., Hazen R.M., McCormick T.C., Prewitt C.T., Smyth J.R. (1988). Comparative compressibility of end-member feldspars. Phys. Chem. Miner..

[41] Fournier F., Leonide P., Biscarrat K., Gallois A., Borgomano J., Foubert A. (2011). Elastic properties of microporous cemented grainstones. Geophysics.

[42] Dvorkin J., Walls J., Davalos G. (2021). Velocity-porosity-mineralogy model for unconventional shale and its applications to digital rock physics. Front. Earth Sci..

[43] Vanorio T., Prasad M., Nur A. (2003). Elastic properties of dry clay mineral aggregates, suspensions and sandstones. Geophys. J. Int..

[44] Prak D.J.L., Lee B.G., Cowart J.S., Trulove P.C. (2017). Density, viscosity, speed of sound, bulk modulus, surface tension, and flash point of binary mixtures of butylbenzene + linear alkanes (n-decane, n-dodecane, n-tetradecane, n-hexadecane, or n-heptadecane) at 0.1 MPa. J. Chem. Eng. Data.

[45] Adam L., Batzle M., Brevik I. (2006). Gassmann’s fluid substitution and shear modulus variability in carbonates at laboratory seismic and ultrasonic frequencies. Geophysics.

[46] Adam L., Batzle M., Lewallen K.T., van Wijk K. (2009). Seismic wave attenuation in carbonates. J. Geophys. Res. Solid Earth.

[47] Mikhaltsevitch V., Lebedev M., Chavez R., Pervukhina M., Glubokovskikh S., Vargas E.A. (2022). The dead volume effect on the elastic moduli measurements using the forced-oscillation method. Geophys. Prospect..

[48] Pimienta L., Borgomano J.V.M., Fortin J., Gueguen Y. (2016). Modelling the drained/undrained transition: Effect of the measuring method and the boundary conditions. Geophys. Prospect..

